# Something New

**Published:** 1877-07-20

**Authors:** 


SOMETHING NEW.—A novel and interesting episode occurred at the late meeting of the American Medical Association. Dr. Andrew McFarland, of Jacksonville, Ill., was married, in the presence of the Association, to Miss Abbie Knox, of St. Louis. The happy pair were almost overwhelmed with the congratulations of the members.

